# The impact of male infertility faculty on urology residency training

**DOI:** 10.1111/and.14457

**Published:** 2022-05-11

**Authors:** Kian Asanad, David Nusbaum, Gerhard Fuchs, John C. S. Rodman, Mary K. Samplaski

**Affiliations:** ^1^ Institute of Urology University of Southern California Los Angeles California USA; ^2^ Keck School of Medicine University of Southern California Los Angeles California USA; ^3^ University of Southern California Southern California Clinical and Translational Science Institute Los Angeles California USA

**Keywords:** in‐service exam, male infertility, microsurgery, resident education, urology resident

## Abstract

The objective of this study was to determine the impact of having male infertility on urology residents' infertility training experience, surgical confidence, and In‐Service‐Exam Infertility/Sexual Medicine subscores. We electronically surveyed urology residents throughout the United States querying exposure to infertility faculty and fertility knowledge. Univariable and multivariable analysis was performed to determine predictors of higher In‐Service Exam Infertility/Sexual Medicine sub‐scores and self‐rated infertility competency. Fifty‐four of 72 respondents (75%) reported that male infertility comprises ≤10% of their training. Of the 63 residents who have a reproductive urologist on faculty, 66.7%, 47.6%, and 49.2% have scrubbed/observed a microsurgical varicocelectomy, vasectomy reversal and testicular sperm extraction, respectively. Residents exposed to infertility faculty are more likely to self‐rate their infertility understanding as “excellent” or “good” (*p* = 0.04 and *p* = 0.02, respectively), and 14.4× more likely to feel confident performing infertility procedures, versus residents lacking faculty (*p* < 0.001). Residents having formal microsurgical training have better self‐rated infertility understanding (*p* < 0.001), non‐obstructive azoospermia management (*p* = 0.01), and competency performing infertility procedures (*p* < 0.001). Residents exposed to fertility faculty are more likely to feel confident performing fertility procedures after residency (*p* = 0.001). In conclusion, infertility comprises a minority of residency training. Most residents anticipate performing infertility procedures in practice, despite two‐thirds lacking confidence performing these. Having an infertility faculty and formal microsurgical training improves residents' surgical confidence, non‐obstructive azoospermia management, and global male infertility understanding. A structured educational curriculum may improve resident infertility training.

## 1 INTRODUCTION

Andrology and male infertility are relatively new urologic subspecialties. The first organized American reproductive medicine meeting was the American Society for the Study of Sterility, in 1944, with 25 attendees (Duka, 1995). In the 1980s, the first American andrology fellowships were established (Krausz et al., 2015), and the number has grown. In 2007, the first Andrology match occurred, four programs participated, and four applicants matched. In 2021, 14 programs are registered for 38 applicants (Association AU, 2021). As the number of male infertility subspecialists has increased, the number of residency programs with fellowship‐trained faculty has also increased.

Despite a growing number of andrology/male infertility trained urologists, the available data suggest a relative lag for resident exposure to this specialty. Data from a resident survey presented at the 2017 American Urological Association (AUA) meeting found that only 36% of institutions had an andrology/male infertility faculty, and 78% of residents reported inadequate andrology/male infertility exposure (Abou Ghayda et al., 2017). Unlike most urologic subspecialties, the Accreditation Council for Graduate Medical Education (ACGME) has not published formalized andrology/male infertility or microsurgery training milestones (Nseyo et al., 2017). Therefore, the male infertility/andrology training for residents is likely still quite variable and some may graduate without adequate exposure to this field.

However, infertility is a topic seen in urologic patient care and tested on In‐Service and board examinations. If training exposure to andrology/male infertility is variable, residents without a reproductive urologist faculty member may be potentially tested on material they have not been exposed to. In the aforementioned 2017 abstract, 77.6% of urology residents stated they would not feel confident getting their own fertility care at their home institution due to competency concerns, demonstrating that many residents graduate without the confidence needed to manage infertility patients.

Complicating matters, andrology training may be different from male infertility training. Male infertility is focused on male reproduction, encompassing hypothalamic–pituitary‐gonadal axis disorders, surgical sperm retrievals, varicocele and vasectomy reversals. Andrology training may include erectile/ejaculatory/sexual dysfunction, Peyronie's disease, priapism, and other conditions. The focus of this manuscript is on male infertility specifically, due to its unique microsurgical, couple‐focused, and financial nuances.

Given the lack of consistent male infertility faculty exposure in urology residency programs, we sought to understand the impact of having a dedicated reproductive urologist (RU) faculty on resident male infertility training. Specifically, we primarily aimed to understand resident self‐perceived competence and confidence in these areas, in programs with and without a dedicated RU. In programs having a dedicated RU, we sought to explore resident exposure to this faculty. We hypothesized that in programs having a dedicated RU, residents would endorse being more confident in medical and surgical management of male infertility patients. We secondarily aimed to identify significant predictors of In‐Service Exam Infertility/Sexual Medicine subscores.

## 2 MATERIALS AND METHODS

### 2.1 Study design

A 26‐question, non‐validated survey was created and designed by the authors of the study, which comprised of multiple choice and Likert scale questions (Figure S1). Questions asked about the presence or absence of a dedicated RU faculty at their residency program, and resident exposure to this faculty, both in clinic and operating suite. Several fertility knowledge‐based questions were included, to determine if knowledge correlated with RU staff presence or exposure. Residents were asked to provide their 2019 In‐Service Exam Infertility/Sexual Medicine subscores. Finally, questions assessing residents' self‐confidence in treating male infertility and future career plans were included. All participants consented to have their responses used for research.

After institutional review board approval, the questionnaire was e‐mailed by our home residency program coordinator to all American Urology Residency Program coordinators, with a request for confidential distribution to all residents. Residents were given 8 weeks from the initial e‐mail date, May 2nd, 2020, until the survey closed. Three reminder emails were sent by our urology residency coordinator.

### 2.2 Statistical analysis

Because of non‐normal data distribution, median and interquartile ranges (IQR) were used to measure central tendency and Wilcoxon Rank Sum testing was used to test for differences between continuous variables (In‐Service scores) and groups. Categorical variables were presented as frequency and column percentages, and Fisher's Exact test or Pearson's Chi‐square was used to test for associations. Exact logistic regression analysis was performed to determine the association between having a RU faculty and resident self‐rated competency. Self‐rated competency was dichotomized (“not competent” and yes “competent”) because of the unbalanced sample in the no RU faculty group. Multivariable linear regression analysis was performed to determine associations between key categorical variables (i.e. RU faculty, percentage residency training in male infertility, prior microsurgical experience) on the 2019 In‐Service Exam Infertility/Sexual Medicine subscores. Potential confounding factors, including resident training year, were controlled for in all final multivariable models. *p* values reported are two‐sided with *p* < 0.05 considered statistically significant. Statistical software R Version 4.0.2 was used for all analyses.

## 3 RESULTS

### 3.1 Cohort characteristics

Seventy‐two residents responded to our survey. Since our internal residency program coordinator sent an email request to all other American urology residency program coordinators, and these outside coordinators may or may not have emailed the survey to their residents, we do not know how many residents actually received the survey, and therefore it is impossible to calculate response rate. It was not required that every question be answered.

A range of resident training years were represented, with 19.4% PGY‐1, 20.8% PGY‐2, 29.2% PGY‐3, 15.3% PGY‐4, 13.9% PGY‐5, and 1.4% PGY‐6. Of the 72 respondents, 77.8% had a fellowship‐trained RU on faculty, 9.7% had a non‐fellowship‐trained RU faculty, and 12.5% did not have a RU faculty. Of those lacking a RU faculty, all felt this would be a beneficial addition to their training. When asked what percentage of training male infertility comprised, 75% indicated 0%–10%, 25% indicated 11%–20%, and none >20%.

In 20.6% of those having a RU (fellowship‐trained or non‐fellowship trained) on staff, the RU had an andrology fellow. Of those having a fellow, 63.5% of residents indicated that the fellow did not affect their learning, 12.7% felt that it enhanced, and 9.5% felt that it detracted from their learning experience.

Most (66.1%) of residents having a RU faculty attended clinic with the RU, and 97.3% found this to be a valuable experience. Forty‐three per cent had some formal microsurgical training, either in a wet‐lab or on patients. Of residents having a RU, 66.7% had scrubbed‐in on a microsurgical varicocelectomy, 47.6% on a microsurgical vasectomy reversal, 19% had seen a percutaneous epididymal sperm aspiration (PESA), and 49.2% had seen a testicular sperm extraction (TESE).

Many RU perform procedures at reproductive endocrine and infertility (REI) fertility clinics for coordination of fertility care. Seventeen per cent of residents knew that their RU performed procedures at REI clinics, but of these only 18.2% had the opportunity to watch or scrub on male procedures performed at these female fertility clinics.

### 3.2 Univariate analysis

Results comparing responses from residents with and without a RU faculty are seen in Table 1. Residents were asked a series of questions, self‐rating their fertility knowledge. Most (63.9%) reported understanding the difference between PESA and TESE, and TESE and microsurgical testicular exploration, and this did not differ between those who did and did not have a RU on faculty (65.1% vs. 55.6% and 68.3% vs. 44.4%, respectively). Understanding of these concepts did not vary by resident clinic exposure to the RU faculty (*p* = 0.08).

**TABLE 1:** Urology resident reported variables affected by presence or absence of a reproductive urologist (RU) on faculty

Variable	*N*	RU on faculty		*p* value
		No (*n* = 9)	Yes (*n* = 63)	
Understand the difference between PESA and TESE?				0.71
No	26	4 (44.4%)	22 (34.5%)	
Yes	46	5 (55.6%)	41 (65.1%)	
Understand the difference between TESE and MicroTESE?				0.26
No	25	5 (55.6%)	20 (31.7%)	
Yes	47	4 (44.4%)	43 (68.3%)	
Exam Sub‐score for infertility^a^	36	58% (3, 83)	66% (15, 100)	0.45
Understanding of male infertility				0.04*
Awful	6	2 (22.2%)	4 (6.3%)	
Poor	13	4 (44.4%)	9 (14.3%)	
Fair	33	3 (33.3%)	30 (47.6%)	
Good	16	0	16 (25.4%)	
Excellent	4	0	4 (6.3%)	
Knowledge in management of NOA				0.55
Awful	6	1 (11.1%)	5 (7.9%)	
Poor	12	3 (33.3%)	9 (14.3%)	
Fair	33	4 (44.4%)	29 (46.0%)	
Good	17	1 (11.1%)	16 (25.4%)	
Excellent	4	0	4 (6.3%)	
Effect exogenous testosterone has on sperm production				0.64
Decrease counts (correct)	64	9 (100%)	55 (87.3%)	
No change (incorrect)	7	0	7 (11.1%)	
Increase counts (incorrect)	1	0	1 (1.6%)	
Feel confident to do procedures after residency training?				0.001*
No	23	8 (88.9%)	15 (23.8%)	
Somewhat	23	1 (11.1%)	22 (34.9%)	
Yes	23	0	23 (36.5%)	

*Note*: Numbers represent frequency (column percentage) and Median (IQR), (min, max) unless otherwise noted. *Significant at *p* = 0.05 level.

^a^ 36 participants were missing Exam Sub‐score.

Residents were asked, “How would you rate your global understanding of male infertility?” There was a significant association between having a RU faculty and self‐rated fertility understanding. Residents having a RU on faculty, or being exposed to this faculty, had a higher proportion of “excellent” and “good” understanding versus those without (31.7% vs. 0%, respectively; *p* = 0.04). Additionally, clinic exposure to the RU faculty was associated with higher self‐rated fund of knowledge for non‐obstructive azoospermia (NOA) management (*p* = 0.049).

Thirty‐six residents provided their 2019 In‐Service Exam Infertility/Sexual Medicine subscore. The median exam subscore was 66% (IQR 33–100). Scores did not differ with presence or absence of a RU faculty, or with exposure to the RU faculty.

A series of questions addressed fund of knowledge. Reassuringly, 88.9% responded that exogenous testosterone would decrease sperm production, and this did not differ between those having a RU on faculty (*p* = 0.64) or having exposure to the RU (*p* = 0.25).

Less than half (39.7%) indicated that having a RU had positively influenced their desire to pursue a male infertility fellowship. Having a RU on faculty did not affect the likelihood of pursuing an infertility fellowship and nor did RU faculty exposure.

Regarding plans to include infertility procedures (vasectomy reversals, sperm retrievals, microsurgical varicocelectomies) in their practice, 80.8% of residents planned to perform infertility procedures “on occasion” or more. However, when asked, “Do you feel competent to do these after residency training is over?” resident responses were evenly split with, 33.3% (23/69) responding ‘yes’, ‘somewhat’, and ‘no’. A significantly higher proportion of residents having a RU on faculty, or exposure to this faculty, felt confident performing fertility procedures after residency (*p* = 0.001 and *p* = 0.001, respectively) (Table 1).

Fisher's Exact test was performed to analyse the association between microsurgical training, and self‐reported knowledge (Table 2). Respondents who had microsurgical training experience in residency had significantly better self‐rated global understanding of male infertility (*p* < 0.001), better knowledge in NOA management (*p* = 0.01), and self‐reported competency to perform infertility procedures after residency (*p* < 0.001).

**TABLE 2:** Association between formal microsurgical training and self‐reported male infertility knowledge and procedural competence

Variable	*N*	Any prior microsurgical training		*p* value
		No (*n* = 31)	Yes (*n* = 41)	
Knowledge in management of NOA				0.01*
Awful	6	5 (16.1%)	1 (2.4%)	
Poor	12	6 (19.4%)	6 (14.6%)	
Fair	33	17 (54.8%)	16 (39.0%)	
Good	17	3 (9.7%)	14 (34.1%)	
Excellent	4	0	4 (9.8%)	
Global understanding of male infertility				<0.001*
Awful	6	5 (16.1%)	1 (2.4%)	
Poor	13	8 (25.8%)	5 (12.2%)	
Fair	33	17 (54.8%)	16 (39.0%)	
Good	16	1 (3.2%)	15 (36.6%)	
Excellent	4	0	4 (9.8%)	
Feel competent to do infertility procedures after residency training?				<0.001*
No	23	19 (61.3%)	4 (9.8%)	
Somewhat	23	8 (25.8%)	15 (36.6%)	
Yes	23	3 (9.7%)	20 (48.8%)	
Exam sub‐scores for infertility^a^	36	75% (15, 85)	66% (3, 100)	0.78
Understand the difference between PESA and TESE?				0.004*
No	26	17 (54.8%)	9 (22.0%)	
Yes	46	14 (45.2%)	32 (78.0%)	
Understand the difference between TESE and MicroTESE?				<0.001*
No	25	18 (58.1%)	7 (17.1%)	
Yes	47	13 (41.9%)	34 (82.9%)	
Effect exogenous testosterone has on sperm production				0.82
Decrease counts (correct)	64	27 (87.1%)	37 (90.2%)	
No change (incorrect)	7	4 (12.9%)	3 (7.3%)	
Increase counts (incorrect)	1	0	1 (2.4%)	

*Note*: Numbers represent frequency (column per cent) and Median (IQR), (min, max) unless otherwise noted. *Significant at *p* = 0.05 level.

^a^ 36 participants were missing exam sub‐score.

### 3.3 Multivariable analysis

Exact logistic regression analysis was performed to examine the association between having a RU faculty and resident self‐rated competency, while controlling for prior training in male infertility. Having a RU faculty was significantly associated with self‐reported competence for performing infertility procedures. Those having a RU on faculty were more likely to feel competent performing male infertility procedures versus those without an RU faculty (OR = 14.4; 95% CI, 2.60‐Inf; *p* value <0.001).

Using multivariable linear regression with in‐service exam score as the outcome (Table 3), after controlling for prior training in male infertility, those that rated their knowledge of NOA management as ‘good’ or ‘excellent’ scored, on average, 49.2 and 65.8 points higher, respectively, on the 2019 In‐Service Exam Infertility/Sexual Medicine section than those that rate their knowledge as ‘awful’ (*p* value = 0.007 and <0.001).

**TABLE 3:** Multivariable linear regression with 2019 in‐service exam infertility/sexual medicine sub‐scores as outcome

Variable	*N*	Estimate	95% confidence interval	*p* value
RU on faculty in program				
No	9	Reference	Reference	
Yes	63	14.96	(−8.25–38.18)	0.196
Per cent of training is in male infertility				
0%–10%	54	Reference	Reference	
11%–20%	18	−11.01	(−26.08–4.06)	0.145
Self‐rated knowledge in management of NOA				
Awful	6	Reference	Reference	
Poor	12	45.25	(4.85–85.65)	0.030*
Fair	33	40.80	(4.60–76.99)	0.029*
Good	17	49.16	(14.65–83.67)	0.007*
Excellent	4	65.79	(34.19–97.38)	<0.001*
Global understanding of male infertility				
Awful	6	Reference	Reference	
Poor	13	−20.99	(−54.60–12.61)	0.210
Fair	33	−9.15	(−40.71–22.41)	0.556
Good	16	−0.62	(−28.97–27.73)	0.965
Excellent	4	NA	NA	NA

*Note*: *Significant at *p* = 0.05 level.

## 4 COMMENT

Male infertility is most often part of andrology training; however, faculty may have predominance for either andrology or male infertility. Subspecialty training in andrology/male infertility is not certified by the American Board of Urology, and thus a relative lack of standardization of curriculum exists for graduating fellows. Fellows matriculating likely have variation in their individual training experiences and bring different skill sets to their faculty positions.

As the number of fellowship‐trained male infertility subspecialists has increased, the number of residency programs with this faculty has also increased. While an AUA andrology core curriculum exists, the ACGME has not published formalized andrology/male infertility milestones for residency programs. Presently, the ACGME requires 40 scrotal/inguinal cases, but there is no specification that these must be andrology or fertility oriented. As a result, male infertility and andrology training for residents is likely still quite variable and some residents may graduate without the confidence needed to manage infertility patients. A review of ABU case logs for certifying urologists found that 9.4% performed at least one vasectomy reversal procedure, suggesting that fertility surgery may be a part of approximately 10% of urologists' armamentarium (Nseyo et al., 2017).

A 2017 abstract found that 63.8% of residents had no andrology/male infertility faculty member, 72% felt their andrology/male infertility knowledge was unsatisfactory, 77.6% felt their training exposure was inadequate, 82.8% had no microsurgical training and 77.6% stated that they would not feel confident getting fertility care at their home institution due to competency concerns (Abou Ghayda et al., 2017). Similar data has been seen in the European literature. A survey of French residents found that 81.8% were interested in a career in andrology, but only 4% felt that their current education was adequate (Nseyo et al., 2017).

This study specifically aimed at understanding American urology resident male infertility exposure and confidence. While there is some overlap between male infertility and andrology, male infertility has some unique aspects, which may make it more difficult for residents to be exposed to training with this faculty, patients or procedures. The focus of this manuscript is on male infertility specifically, due to its unique microsurgical, couple‐focused, and financial nuances.

Infertility is the only urologic specialty that requires communication with the female partners' REI. Sperm retrieval procedures may be done at REI‐based fertility clinics, where an andrologist or embryologist is on‐site to check samples in real‐time so that additional samples may be taken, tailored to the number of eggs available. Given competing academic commitments, including multiple staff and hospitals/clinics covered, and credentialing needs, urology residents may not be able to actually attend these sperm retrieval procedures despite having a RU faculty. Our data reflected this pattern. Less than 20% of residents had the opportunity to watch or scrub in on male infertility procedures performed at female fertility clinics. Likewise, only 19% had even seen a PESA during their training. This is in agreement with a recent study evaluating and comparing male infertility exposure among residents between the United States and Canada. Similarly, the majority of the respondents (80%) their infertility exposure during residency was inadequate and less than 20% of programs offered formal microsurgical training (Ghayda et al., 2021).

Outside of paediatric urologists occasionally integrating a microscope for hypospadias repair, male infertility is the main urologic sub‐specialty utilizing the operative microscope, for vasectomy reversals, varicocelectomies, epididymal sperm aspirations and testicular sperm extractions (among others). The coordination, manual dexterity and steadiness needed for microsurgery requires training and practice to achieve. Success in these procedures is highly dependent on microsurgical proficiency, and the stakes for patients are high (Mehta & Li, 2013). Presently, there are no ACGME microsurgical requirements for graduating urology residents. This is reflected in our data, where we found that only 43% of residents had formal microsurgical training, either wet‐lab or on patients. Similarly, less than half of residents had scrubbed on a microsurgical vasectomy reversal. If our future urologists will be performing these operations, as 72.2% of residents anticipated doing occasionally or more, it is vital that they have exposure to this type of training. On multivariate analysis, having a RU on faculty was significantly associated (OR = 14.4) with feeling competent performing male infertility procedures. Thus, we argue that implementing a structured educational curriculum for male infertility and microsurgery training may standardize, and indeed optimize, resident education in male infertility.

Male infertility is also unique in that many of the procedures and visits are cash pay. This may drive patients to be seen at satellite clinics or surgical centers with the least amount of overhead, but without resident coverage. In addition, some patients may ask that trainees not be involved in their procedures, for fear that trainees may impair outcomes. Many RU also have fellows. We found that while 20% of RU's had fellows, reassuringly 76% of residents reported that the fellow either did not affect or enhanced their learning.

Our study has limitations. The survey used was not validated. The available validated educational questionnaires would not have gathered the information we sought to understand. The survey was not designed in accordance with the Checklist for Reporting Results of Internet E‐Surveys (CHERRIES) (Eysenbach, 2004). Although the CHERRIES checklist specifically focuses on web‐based surveys administered on the Internet, rather than distributed via electronic mail, many of the CHERRIES items are valid for our study. This includes survey design, IRB exemption, survey administration, and analysis. In addition, we do not know how many residents received questionnaires, and therefore we are unable to calculate response rate. However, we may assume the response rate is low given we received 72 responses and there are 365 residency positions in the country per year. Finally, for some questions, there was a degree of non‐response, making the data set incomplete.

## 5 CONCLUSIONS

Male infertility comprises a small percentage of American urology residency training. Less than half of residents were exposed to fundamental microsurgical procedures such as vasectomy reversals and TESEs, despite the majority (80%) anticipating performing infertility procedures in their future practice. Having a RU on faculty, and exposure to this faculty, is associated with higher resident self‐competence in male infertility procedures.

## CONFLICT OF INTEREST

The authors declare no conflict of interest.

## Supporting information


Supplementary Figure 1

## Data Availability

Data sharing is not applicable to this article as no new data were created or analyzed in this study.
