# Supplementary material for: The impact of male infertility faculty on urology residency training
Source: Andrologia. 2022 May 11;54(8):e14457. doi: 10.1111/and.14457 (PMC9540376; doi:10.1111/and.14457)
Supplement: Supplementary file 1 — Supplementary Figure 1 [file AND-54-e14457-s001.docx]

*COHORT CHARACTERISTICS*

**Q1. Do you have a Reproductive Urologist on faculty in your program? (defined as a provider with component of their practice involving the treatment of male infertility patients)**

- 1. Yes, fellowship-trained
  2. Yes, but not fellowship-trained
  3. No

**Q2. If “no”, do you believe that this would be a beneficial part of your training?**

1. Yes
2. No

**Q3. Does your Reproductive Urologist have a fellow?**

1. Yes
2. No

**Q4. Does the fellow impact your fertility learning experience?**

1. Yes, enhances my learning experience
2. Yes, detracts from my learning experience
3. No, does not impact my learning experience

**Q5. Do you attend clinic with your Reproductive Urologist?**

1. Yes
2. No
3. N/A, no Reproductive Urologist on faculty

**Q6. If you do go to clinic with your Reproductive Urologist, do you find this to be a valuable learning experience?**

1. Yes
2. No
3. N/A, no Reproductive Urologist on faculty

*PROCEDURAL EXPERIENCE*

**Q7. Have you had any formal microsurgical training (on patients or a wet lab) during your residency?**

1. Yes
2. No

**Q8. Have you scrubbed in on a vasectomy reversal with your Reproductive Urologist?**

1. Yes
2. No
3. N/A, no Reproductive Urologist on faculty

**Q9. Have you scrubbed in on a microsurgical varicocelectomy with your Reproductive Urologist?**

1. Yes
2. No
3. N/A, no Reproductive Urologist on faculty

**Q10. Have you seen a percutaneous epididymal sperm extraction (PESA) for sperm retrieval with your Reproductive Urologist?**

1. Yes
2. No
3. N/A, no Reproductive Urologist on faculty

**Q11. Have you seen a testicular sperm extraction (TESE) for sperm retrieval with your Reproductive Urologist?**

1. Yes
2. No
3. N/A, no Reproductive Urologist on faculty

**Q12. Do you understand the difference between a PESA and a TESE?**

1. Yes
2. No

**Q13. Do you understand the difference between a TESE and a microTESE?**

1. Yes
2. No

**Q14. Does your Reproductive Urologist perform procedures at female fertility clinics (for coordination of patient care)?**

1. Yes
2. No
3. I do not know

**Q15. Have you had the opportunity to watch or scrub in on male infertility procedures performed at female fertility clinics?**

1. Yes
2. No
3. N/A

*GLOBAL ASSESSMENT*

**Q16. What percentage of your residency training does male infertility comprise?**

1. 0-10%
2. 11-20%
3. 21-30%
4. 31-40%
5. 41% or more

**Q17. How would you rate your global understanding of male infertility?**

1. Excellent
2. Good
3. Fair
4. Poor
5. Awful

*KNOWLEDGE-BASED QUESTIONS*

**Q18. What was your 2019 Urology in-service exam sub-score for Infertility/Sexual Medicine (please refer to your exam scoring results)? _____**

**Q19. How would you rate your fund of knowledge in the management of nonobstructive azoospermia?**

1. Excellent
2. Good
3. Fair
4. Poor
5. Awful

**Q20. Is an ejaculate volume of 0.5cc??**

1. Lower than normal
2. In normal range
3. Higher than normal

**Q21. What effect will exogenous testosterone (testosterone gel, testosterone cypionate) have on sperm production?**

1. Increase sperm counts
2. Decrease sperm counts
3. No change in sperm parameters

*FUTURE IMPACT*

**Q22. Has the presence of a Reproductive Urologist on faculty in your program influenced your desire to pursue a fellowship in male infertility?**

1. Yes, more interested in male infertility fellowships
2. Yes, less interested in male infertility fellowships
3. No, I found a different career path independent of Reproductive Urologist exposure

**Q23. Do you plan to do male infertility procedures (vasectomy reversals, sperm retrievals, microsurgical varicocelectomies) in your practice?**

1. Yes, a large part of my practice (>70%)
2. Yes, some of my practice (30-70%)
3. On occasion, as needed (10-20%)
4. Rarely to never (10% or less)

**Q24. If you do plan to do male infertility procedures, do you feel competent to do these after residency training is over?**

1. Yes
2. Somewhat
3. Not

*OTHER*

**Q25. What year in training are you?**

1. PGY-1
2. PGY-2
3. PGY-3
4. PGY-4
5. PGY-5
6. PGY-6

**Q26. What state is your residency located? _____**
